# Variations in the association between standalone and coexisting forms of undernutrition and common infectious morbidities among children in sub-Saharan Africa

**DOI:** 10.1016/j.ghrp.2026.06.004

**Published:** 2026-06-11

**Authors:** Misganaw Gebrie Worku, Theo Niyonsenga, Zelalem Mengesha, Itismita Mohanty

**Affiliations:** a Health Research Institute, Faculty of Health, University of Canberra, Canberra 2617, Australia; b Department of Human Anatomy, School of Medicine, College of Medicine and Health Sciences, University of Gondar, Gondar, Ethiopia

**Keywords:** Undernutrition, Coexisting undernutrition forms, Infectious morbidity, Children, Composite index of anthropometric failure

## Abstract

**Background:**

Undernutrition is a major driver of common infectious morbidity among children under five; however, the relationship between different forms of undernutrition and childhood infectious morbidity remains poorly understood. This study examined variations in the association between different forms of undernutrition measured according to the Composite Index of Anthropometric Failure (CIAF) and common infectious morbidity among children under the age of five in sub-Saharan Africa (SSA).

**Methods:**

We performed a multilevel binary logistic regression analysis using country and community clusters as random effects. Our study utilised demographic and health survey (DHS) data collected between 2016 and 2024 in 27 SSA countries. A total weighted sample of 157, 800 under-five children whose nutritional status was assessed based on the World Health Organization (WHO) anthropometric techniques and data on Acute Respiratory tract Infection (ARI) and diarrhea recorded were included. An adjusted odds ratio (AOR) with a 95% Confidence Interval (CI) was reported, and variables’ effects with a *p*-value less than 0.05 were declared significant determinants of common infectious morbidity.

**Results:**

The prevalence of common infectious morbidity among children under five in SSA was 30.20% (95% CI: 27.34, 33.06). The lowest and highest prevalences were reported in Mozambique (16.96%; 95% CI: 16.94, 16.98) and Uganda (53.26%; 95% CI: 53.24, 53.28), respectively. The odds of infectious morbidity significantly differs between children with standalone, double and triple forms of undernutrition. Children with double (AOR: 1.25; 95% CI: 1.16, 1.34 for stunting-underweight; AOR: 1.36; 95% CI: 1.22, 1.51 for wasting-underweight) and triple undernutrition (AOR: 1.51; 95% CI: 1.36, 1.68) were more susceptible to common infectious morbidity.

**Conclusions:**

Children with coexisting undernutrition were more likely to experience common infectious morbidity, and those affected by the coexistence of stunting-wasting-underweight experienced the highest odds of infectious morbidity. Among the standalone forms, only underweight children were more likely to experience common infectious morbidity. Therefore, to mitigate the burden of childhood infectious morbidity, it is crucial for policymakers to implement targeted nutritional interventions for children experiencing coexisting undernutrition.

## Background

Communicable diseases continue to present a significant public health challenge in low- and middle-income countries (LMICs).^1^, ^2^ Acute respiratory tract infection (ARI) and diarrhea are the two most common childhood infectious problems, accounting for 29% of all child deaths globally.^3^, ^4^ ARI is the third leading cause of child mortality (account for 5.8 million deaths) globally and is the primary cause of child death in LMICs.^5^, ^6^ In SSA, approximately one in four children experiences ARI.^1^, ^5^ Similarly, diarrhea is the second leading cause of child mortality in LMICs, contributing to 525,000 deaths annually.^7^ Recurrent diarrhea in children leads to growth retardation and impaired cognitive function.^3^, ^8^ This implies that ARI and diarrhea are the leading infectious conditions impacting the growing children.

In response to these public health challenges, the World Health Organization (WHO) and United Nations International Children's Emergency Fund (UNICEF) introduced the integrated Global Action Plan for the Prevention and Control of Pneumonia and Diarrhea (GAPPD).^1^, ^9^ Since pneumonia and diarrhea share similar determinants, preventive strategies and interventions are often implemented through healthcare facilities, communities, and schools in a comparable manner. The GAPPD action plan prioritized children with poor nutrition, low socioeconomic status, and those from remote areas, as these groups are most at risk of dying from pneumonia and diarrhea.^3^ Improving child nutrition is the foremost GAPPD strategy to safeguard children from these diseases and the associated mortality.

Child nutritional status plays a significant role in determining susceptibility to childhood infectious morbidity, as being undernourished weakens immune defences.^10^, ^11^ Nearly 44% of ARI and 28% of diarrhoea cases among children are attributed to poor nutrition.^12^ On the contrary, literature also supports that infectious diseases could lead to poor nutrition through impairing nutrient absorption, causing loss of appetite, and essential nutrients.^13^ The immune activation associated with infectious diseases also depletes the body's energy and leads to nutrient deficiency.^14^ In essence, the burden of child undernutrition can be significantly influenced by infectious disease.^15^, ^16^ This bidirectional association between undernutrition and common infectious diseases encompasses various basic and underlying factors.^17^, ^18^, ^19^ However, in this study, we conceptualized a unidirectional relationship from undernutrition to infectious morbidity, viewing undernutrition as a risk factor due to its chronic nature. In contrast, common infectious conditions such as ARI and diarrhea, as captured in the demographic health survey (DHS), are typically acute in onset. This distinction is influenced by the structure of the DHS questionnaire, which records infectious morbidity based on a two-week recall period. Therefore, the temporality and nature of the data support the assumption that undernutrition precedes and potentially contributes to the occurrence of these infections.

Extensive literature has highlighted a noteworthy connection between undernutrition, childhood infectious morbidity, and associated deaths among children.^20^, ^21^, ^22^ Most of these studies have relied on conventional undernutrition indicators (stunting, wasting and underweight as an independent measure of undernutrition) to estimate the risk of morbidity and mortality associated with undernutrition. However, in this study, we argue that the conventional undernutrition measurements provide limited information on the relationship between undernutrition and comorbidity conditions. This is because these measures obscure children who are simultaneously affected by combinations of stunting, wasting and/or underweight, which are considered the most vulnerable groups by other studies.^23^, ^24^, ^25^ The Composite Index of Anthropometric Failure (CIAF) is a comprehensive measure of undernutrition that captures all forms of anthropometric failure, addressing the limitations of conventional indicators.^26^ Unlike traditional approaches, CIAF provides non-overlapping categories of undernutrition, offering a more accurate assessment.^23^, ^27^ It is constructed by combining conventional anthropometric indicators: stunting, wasting, and underweight into a single nutritional index at the population level.^27^ CIAF can be used in both aggregated and disaggregated forms to estimate the overall burden of undernutrition and to analyse patterns among specific subgroups.^28^ The aggregated CIAF, which includes any child with at least one anthropometric failure, offers a broad view of the total undernutrition burden. In contrast, the disaggregated CIAF distinguishes between children with single (stunting only, wasting only, underweight only), double (stunting + underweight, wasting + underweight), and triple (stunting + wasting + underweight) forms of undernutrition, providing more detailed insight.^29^

Few studies that applied the CIAF approach have shown that children with double and triple undernutrition experience a higher risk of diarrhea and ARI than children with standalone forms of undernutrition.^24^, ^26^, ^29^, ^30^ However, these studies did not adjust for other confounding factors, including sociodemographic and socioeconomic factors that may affect common childhood infectious morbidity, which could potentially lead to biased results. Another study compared the risk of infectious morbidity using both conventional methods and the CIAF approach.^24^ The conventional methods did not allow to pinpoint any significant association between any form of undernutrition and the risk of common infectious morbidity. However, when the disaggregated CIAF was applied to the same population, children with coexisting undernutrition experienced higher risks of ARI and diarrhea. This indicates that CIAF is a more appropriate approach for illustrating the risk of common infectious morbidity among children compared to the conventional approach. Therefore, this study aims to investigate variations in the association between various standalone and coexisting forms of undernutrition and common infectious morbidity among under-five children in SSA using CIAF.

## Methods

### Study design

**Fig. 1:**
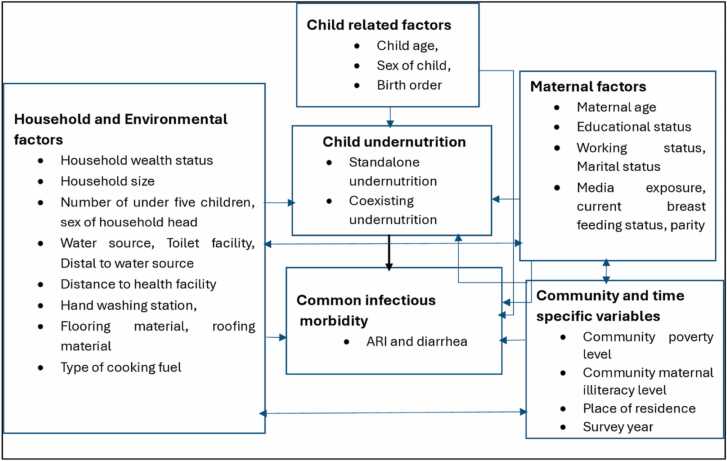
The conceptual framework depicts covariates associated with common infectious morbidity (acute respiratory tract infection and/or Diarrhoea) among children. **Note**: ARI: Acute respiratory tract infection, Standalone undernutrition: children experienced only one form of undernutrition, Coexisting undernutrition: children experienced more than one form of undernutrition simultaneously.

This study involves quantitative analysis of DHS data collected based on cross-sectional design. DHS Data from multiple SSA countries, a region located south of the Sahara Desert, was included. DHS are nationally representative conducted using standard methodology and pretested validated tools.^31^ A standardised protocol was designed for each target group such as children, women, and men at the household level within countries. The DHS provides data on essential health indicators such as morbidity, mortality, family planning, child nutrition and health service utilisation. The study population was children aged under five years in the sampled DHS clusters. Children with nutrition status reported according to the WHO growth standards were included to investigate association between undernutrition and common infectious morbidities while adjusting for confounders (Fig. 1).

DHS data from 27 countries in SSA conducted between 2016 and 2024 was used. Considering the united nation (UN's) decades of action on nutrition (2016–2025) and the commencement of sustainable development goals (SDGs), countries with data collected during recent phase of DHS (phase VII and VIII) were included. This could make our findings more relevant to current global nutrition policy frameworks. Also including recent DHS ensures consistency in the data collection tools used and availability of key covariates across survey years. The DHS data for different target populations are organized in different records such as household recode, women recode, child or kids recode, birth recode and men recode. For this study, kids recode dataset containing information on under-five children was used. The dataset included a 157,800 weighted sample of under-five children.

Regarding sample selection, a two-stage stratified probability sampling was implemented. The DHS sample is stratified by geographical region and place of residence within regions. The survey uses an existing population and housing census frame to draw representative samples from participating countries. In the first stage, a fixed amount of enumeration areas (EA) was selected based on probability of proportional allocation. Household listing was then conducted in the selected EA. In the second stage, a fixed number of households per enumeration area were selected using a systematic selection criterion. All women aged 15–49 years and men aged 15–59 years in the selected household were then considered for the interview. This multistage cluster sampling in DHS leads to hierarchical data nature. Observations have been nested in levels where individual was nested in household, household nested in enumeration areas and enumeration areas nested in country.

### Outcome variable

The outcome variable in this study was common infectious morbidity among children under five. The DHS has recorded common childhood infectious illnesses such as ARI and diarrhea in its survey. Under-five children were considered as having infectious morbidity if they experienced at least one of the two common childhood illnesses (ARI and/or diarrhea) and were categorised as “Yes.” In contrast, those who did not experience any of these illnesses were classified as “No”. In DHS, the presence of ARIs was defined as a child with a history of cough within two weeks preceding the survey, accompanied by short and rapid breathing. Diarrhea among children in the DHS was diagnosed based on whether the child experienced three or more loose or liquid stools per 24-hour period in the 2 weeks preceding the survey.^31^

### Independent variables

The DHS dataset contains child and mother-specific information on their health service utilisation, sociodemographic characteristics, and environment-related data.^32^ Initially, we considered all child, mother, health service, household, and environment-related factors as determinants of common infectious morbidity.^32^ However, variables related to child feeding, childcare, and health service utilization were excluded during preliminary analysis, as these were collected only for children under 24 months.^31^ Since the study focused on children aged 0–59 months, restricting the analysis to variables available across the entire age group ensured consistency.

The primary exposure variable in this study was undernutrition among under-five children, defined by using the CIAF. Although the DHS cross-sectional design presents challenges in establishing causal direction, undernutrition was treated as the exposure variable, given its chronic nature. This assumption is further supported by the nature of the DHS data, which recorded common infectious morbidity based on a recent two-week recall period, suggesting that undernutrition likely preceded the onset of infectious morbidity. Anthropometric measurements such as stunting (height-for-age z-scores), underweight (weight-for-age z-scores), and wasting (weight-for-height z-scores) were recorded in accordance with WHO growth standards. The DHS collect data on child nutritional status based on the conventional indicators such as stunting, wasting, and underweight. To capture the complexity of nutritional failure, we disaggregated the three conventional indicator into mutually exclusive categories: standalone (stunting only, wasting only, and underweight only), double (stunting-underweight and wasting-underweight), and triple (stunting-wasting-underweight) forms.^33^ This disaggregated classification enabled us to examine how different combinations of undernutrition influence the risk of common infectious morbidity.

Confounders considered in this study were selected based on their established associations with undernutrition and infectious morbidity^10^, ^24^, ^33^, ^34^ and availability in the DHS dataset.^31^, ^32^ The following covariates were considered in the model: child age in months, sex of the child, birth order, maternal age, maternal educational level, maternal working status, current breastfeeding status, sources of drinking water, type of toilet facility, distance to water sources, distance to health facility, household wealth status, type of cooking fuel, flooring and roofing material, presence of hand washing station in the household, sex of household head, mother’s media exposure status, place of residence, survey year, community illiteracy and community poverty level. Controlling for this set of covariates, which are linked to both undernutrition and infectious morbidity, allows for the proper estimation of the true association between undernutrition and common infectious morbidity.

The conceptual framework, presented in Fig. 1 below, was informed by previous literature identifying risk factors of undernutrition and common infectious morbidity.^33^, ^35^, ^36^, ^37^ It outlines potential confounding factors that may influence the observed association between undernutrition and infectious morbidity, while also illustrating possible bidirectional relationships among the confounders themselves. Although the framework acknowledges interrelationships among certain covariates, these variables were not considered as mediators of association between the exposure (undernutrition) and the outcome (common infectious morbidity). Multicollinearity among variables was assessed and addressed prior to model fitting. Importantly, the framework assumes a unidirectional association from undernutrition to common infectious morbidity. This assumption is based on the chronic nature of undernutrition in our study and the fact that DHS captures infectious morbidity data over a recent two-week recall period (Fig. 1).

### Data management and statistical analysis

All the analyses were conducted based on the weighted data. Data management and analysis were performed using STATA 18 software. Both descriptive and analytical analyses were conducted. Both bivariable and multivariable multilevel binary logistic regression analyses were conducted. Variables with a *p*-value less than 0.2 in the bivariable analysis were included in the multivariable model. In the multivariable analysis, variables with a *p*-value of < 0.05 were considered statistically significant determinants of common infectious morbidity. Finally, the adjusted odds ratios (aOR) with associated 95% confidence interval (CI) were reported as effect measures.

As the DHS data is hierarchical, a multilevel analysis was more appropriate to obtain a more accurate estimate of the model parameters and avoid parameter overestimation. Thus, a multilevel binary logistic regression was implemented to examine the associations between under-five undernutrition and common childhood infectious morbidity.

Multicollinearity among the included covariates was checked. A high-variance inflation factors (VIF) were observed for parity and birth order. At the same time, the VIF value was less than 3 for all other covariates. Thus, parity was excluded from the regression model since it is highly correlated with birth order. To assess and estimate the clustering effect in the outcome between countries, community clusters, and households, we initially considered country, community cluster, and household as random effects. However, the sample size per household was too small with most households containing only one observation. As a result, we excluded households from the random intercepts. Therefore, the multilevel regression model included country and community clusters as random intercepts. We also used a random effect meta-analysis to derive the pooled prevalence of common infectious morbidity in SSA using country level prevalence data of common infectious morbidity.

The Intraclass Correlation Coefficient (ICC), Proportional Change in Variance (PCV), and Median Odds Ratio (MOR) were calculated to evaluate clustering of the outcome across different levels of data hierarchy. In this study, four models were fitted: the null-model, a model with the random intercepts only excluding the explanatory variable; Model 1-a model where child and maternal related explanatory variables added to the null-model; Model 2-a model with household factors added to the model−1; and Model 3-a model with community and time specific variables added to the model−2. The final results were interpreted based on the best-fitting model, determined using the log-likelihood ratio, Akaike’s Information Criterion (AIC), and Bayesian Information Criterion (BIC).

We also assessed the interaction of the exposure variable (undernutrition) and selected covariates (maternal education, maternal working status, wealth status and household and environmental factors) with the survey year to assess any interaction effect of undernutrition and other covariates on common infectious morbidity. This is because we assumed that differences in the outcome and exposure variables might occur across DHS survey years. However, non-significant interaction effects of the year of the survey and undernutrition and year of the survey and other socioeconomic and sociodemographic covariates were observed. Consequently, we excluded the interaction terms from the final model.

Lastly, given the cross-sectional nature of the DHS data and to explore the possibility of reverse association-specifically, whether common infectious morbidity contributes to undernutrition-we calculated the residuals of undernutrition and examined their association with common infectious morbidity. The residuals, representing the unexplained components of undernutrition after adjusting for relevant covariates, were not significantly associated with common infectious morbidity. This lack of association suggests that reverse association is unlikely, as the model adequately controlled for potential confounders.

## Results

### Characteristics of the study participants

Table 1 illustrates the baseline characteristics of the study participants. Among the 157,800 weighted sample of under-five children, 20.87% and 22.11% were aged 12-23 and 0-11 months, respectively. Over half (50.64%) of under-five children were male. Overall, 34.68% of under-five children in SSA were undernourished. Of these, 17.43% and 10.11% experienced stunting only and coexisting stunting-underweight, respectively. Additionally, 50.12% and 48.91% of children had access to improved water and toilet facilities, respectively. A handwashing station was observed among 71.91% of households for use by household members.

**Table 1:** Characteristics of under-five children in sub-Saharan Africa.

**Independent variable**	**Categories**	**Frequency**	**Percentage (weighted)**
**Child-related characteristics**			
Undernutrition	Nourished	104,415	65.32
	Stunting only	27,547	17.43
	Wasting only	3292	1.94
	Underweight only	2083	1.19
	Stunting-underweight	16,461	10.11
	Wasting-underweight	3555	2.07
	Stunting-wasting-underweight	3323	1.95
Sex of child	Male	81,268	50.64
	Female	79,408	49.36
Child age in month	0–11	35,566	22.11
	12–23	33,514	20.87
	24–35	30,892	19.10
	36–47	31,086	19.56
	48–59	29,618	18.37
Birth order	First	35,210	22.22
	2nd−4th	78,899	49.57
	5th and above	46,567	28.20
**Maternal-related characteristics**			
Marital status	Not married	20,566	12.56
	Married/living with partner	140,110	87.44
Maternal age	15–24	44,140	27.00
	25–34	76,845	48.50
	35–49	39,691	24.50
Maternal educational status	No	62,889	37.75
	Primary education	51,248	32.05
	Secondary	46,539	30.20
	Higher	7005	5.09
Maternal working status	Not working	68,372	41.48
	Working	9,2304	58.52
Current breast feeding status	No	69,092	43.25
	Yes	91,584	56.75
Parity	Primiparous	24,637	15.71
	Multiparous	83,083	52.22
	Grand multiparous	52,956	32.08
Media exposure	No	57,917	34.23
	Yes	102,756	65.77
**Household and environmental related characteristics**			
Water source	Not improved	81,771	49.88
	Improved	78,905	50.12
Toilet facility	Not improved	85,525	51.09
	Improved	75,151	48.91
Distance to the health facility	Not a big problem	91,066	38.79
	Big problem	59,591	61.21
Distance to water sources	On-premises	48,651	32.80
	30 min and less	79,150	49.99
	Greater than 30 min	28,534	17.21
Type of cooking fuel	Unclean fuel	138,008	84.83
	Clean fuel	19,672	15.17
Sex of household head	Male	124,415	78.38
	Female	36,261	21.62
Flooring material	Not improved	77,433	47.96
	Improved	80,400	52.04
Roofing material	Not improved	40,083	23.69
	Improved	117,750	76.31
Number of under five children in the household	Less than 3	117,515	74.39
	3 and more	43,161	25.61
Household size	4 and less	39,134	25.23
	5–9	91,949	57.23
	10 and above	29,593	17.54
Hand washing station	Not observed	43,744	28.09
	Observed	116,932	71.91
Household wealth status	Poorest	42,492	22.68
	Poorer	34,223	21.20
	Middle	32,370	20.21
	Richer	28,030	19.10
	Richest	23,561	16.82
**Community and time-specific characteristics**			
Place of residence	Rural	107,440	65.34
	Urban	53,236	34.66
Community illiteracy level	Low	105,950	70.41
	High	54,726	29.59
Community poverty level	Low	80,165	53.55
	High	80,511	46.45
Year of survey	Before covid	90,154	57.09
	During covid	70,522	42.91

### Magnitude of common infectious morbidity in sub-Saharan Africa

**Fig. 2:**
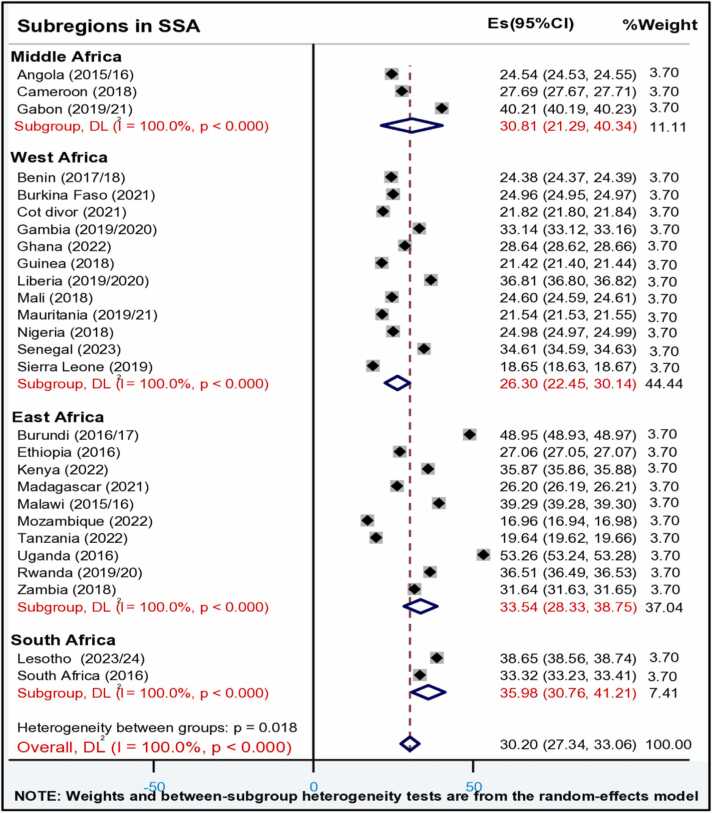
Forest plot showing the magnitude of common infectious morbidity in sub-Saharan Africa.

The forest plot from the meta-analysis, which utilised the individual country level estimates of common infectious morbidity to estimate the pooled prevalence of common infectious morbidity in SSA (presented in Fig. 2) revealed, the pooled estimate was 30.20% (95% CI: 27.34, 33.06, with heterogeneity of I²(I-squared) = 100, *p*-value < 0.001). The prevalence ranges from 16.96% (95% CI: 16.94, 16.98) in Mozambique to 53.26% (95% CI: 53.24, 53.28) in Uganda. The highest and lowest prevalences of common infectious morbidity were reported in South Africa (35.98%; 95% CI: 30.76, 41.21, I² = 100, *p*-value < 0.001) and West Africa (26.30%; 95% CI: 22.45, 30.14, I² = 100, *p*-value < 0.001), respectively.

**Fig. 3:**
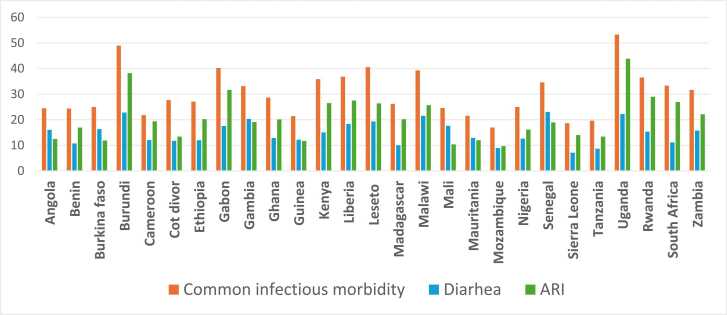
Prevalences of common infectious morbidity, diarrhea, and ARI in sub-Saharan Africa **Note:** ARI: Acute respiratory tract infection, common infectious morbidity: children affected by either diarrhea, ARI, or both diarrhea and ARI simultaneously.

Additionally, 14.67% (95%CI: 14.49, 14.84) and 19.97% (95%CI: 19.77, 20.17) of under-five children in SSA experienced diarrhea and acute respiratory infection, respectively. The highest prevalence of ARI was reported in Uganda (43.81%; 95% CI: 42.34, 45.30) while the lowest was in Mozambique (9.69%; 95% CI: 8.80, 10.67). Similarly, the highest prevalence of diarrhea was reported in Senegal (23.08%; 95%CI: 21.83, 24.37) and the lowest in Sierra Leone (7.13%; 95%CI: 6.37, 7.97) (Fig. 3).

### Random effect analysis and model’s goodness of fit

The random effect model was assessed using variance and ICC. The total ICC value in the null model was 0.05 at the country level and 0.19 at the community level, indicating that 5% and 19% of the total variation in common childhood infectious morbidity were attributable to differences in country and community clusters. Additionally, the high MOR value at the country (1.22) and community (1.75) levels indicates significant differences in the odds of common infectious morbidity between country and community clusters. The MOR value of 1.22 at the country level suggests that when two individuals were selected randomly from two different countries, individuals from the high-risk country were 1.22 times more likely to experience common infectious morbidity. Likewise, if we choose two children from two different community clusters, children from clusters with a higher risk of common infectious morbidity had 1.75 times higher odds of experiencing it than those from clusters with a low risk of common infectious morbidity. Moreover, the included individual, household, and community-level factors explained nearly 19.40% of variations in common infectious morbidity. Regarding the model’s goodness of fit and comparison, the final model (Model−3 incorporating individual, household, and community level factors) was the best-fit model for the data since it had the lowest deviance, AIC, and BIC (Table 2, Table 3).

**Table 2:** Random effect analysis and model fit for assessing the effects of undernutrition on common infectious morbidity.

**Random effect analysis and model fit**					
**Random component**		**Null model**	**Model I**	**Model II**	**Model III**
**Variance**	Country	0.21	0.21	0.19	0.18
	Community	0.59	0.56	0.56	0.56
**ICC**	Country	0.05	0.05	0.05	0.04
	Community	0.19	0.19	0.19	0.18
**MOR**	Country	1.22(1.12, 1.39)	1.22(1.13, 1.39)	1.19(1.12, 1.33)	1.19(1.12, 1.33)
	Community	1.75(1.70, 1.80)	1.70(1.58, 1.82)	1.70(1.58, 1.85)	1.70(1.58, 1.85)
**PCV**	Country	Ref	-	9.52%	14.3%
	Community	Ref	5.1%	5.1%	5.1%
**Model comparison**					
**Log-likelihood**		−89840.238	−89023.491	−81107.741	−81106.376
**Deviance**		179680.476	178046.982	162215.482	162212.752
**AIC**		179686.5	178091	162269.5	162264.8
**BIC**		179716.4	178310.7	162536.6	162522

**Note:** AIC: Akaike’s information criteria; BIC: Bayesian information criterion; ICC: Intraclass Correlation Coefficient; MOR: Median Odds Ratio, N/A: Not applicable, OR: Odds ratio, PCV: Proportional Change in Variance

### Determinants of common infectious morbidity in sub-Saharan Africa

Under-five children experiencing the coexistence of stunting-wasting-underweight were 1.51 times (AOR: 1.51; 95% CI: 1.36, 1.68) more likely to suffer common infectious morbidity than nourished children. Regarding double forms of undernutrition, children with wasting-underweight were 1.36 times (AOR: 1.36; 95% CI: 1.22, 1.51) more likely to have common infectious morbidity compared to the nourished children. Additionally, children with coexisting stunting-underweight were 1.25 times (AOR: 1.25; 95% CI: 1.16, 1.34) more likely to be affected by common infectious morbidity compared to well-nourished children. Regarding standalone undernutrition forms, children experiencing only underweight had 1.28 times (AOR: 1.28; 95% CI: 1.13, 1.46) higher chance of experiencing common infectious morbidity. The findings of this study emphasis that the coexisting manifestations of child undernutrition significantly contribute to the high burden of common infectious morbidity in SSA.

Age of the child was another statistically significant determinant of common infectious morbidity among under-five children. Children aged 12–23 months had 1.30 times (AOR: 1.30; 95% CI: 1.22, 1.38) higher odds of common infectious morbidity. In contrast, children aged above 24 months showed a reduced odd of common infectious morbidity: AOR: 0.81 (95% CI: 0.75, 0.87) for 24–35 months, AOR: 0.59; (95% CI: 0.54, 0.64) for 36–47 months, AOR: 0.45 (95%CI: 0.41, 0.50) for 48–59 months.

Moreover, maternal age of 25–34 (AOR: 0.93, 95% CI: 0.88, 0.96) and 35–49 (AOR: 0.85, 95% CI: 0.80, 0.89) years were associated with a reduced odd of common infectious morbidity compared to maternal age of 15–24 years. Children of working mothers had 1.21 times (AOR: 1.21; 95% CI: 1.15, 1.29) higher odds of common infectious morbidity. The odds of common infectious morbidity among current breastfeeding mother was low compared to their counterpart (AOR: 0.81; 95%CI: 0.78, 0.84). In SSA, media exposure was also identified as a risk factor for common infectious morbidity, with children of mothers with media exposure had 1.23 times (AOR: 1.23, 95% CI: 1.17, 1.29) higher chance of common infectious morbidity (Table 3).

**Table 3:** A multilevel mixed effect analysis illustrating the association between disaggregated patterns of the composite index of anthropometric failure and common childhood infectious morbidity, adjusted for other covariates.

**Independent variables**	**Categories**	**Bivariate analysis (Unadjusted odds ratio)**	**Model I (child-maternal characteristics** **Adjusted odds ratio**	**Model II (child-maternal-household characteristics)** **Adjusted odds ratio**	**Model III (child-maternal-household-community-time specific characteristics)** **Adjusted odds ratio**
**Undernutrition**	Well-nourished	Ref	Ref	Ref	Ref
	Stunting only	1.01(0.97, 1.06)	1.01(0.96, 1.06)	1.01(0.96, 1.06)	1.01(0.96, 1.06)
	Wasting only	0.99(0.89, 1.10)	0.98(0.89, 1.08)	0.98(0.89, 1.08)	0.97(0.88, 1.08)
	Underweight only	1.24(1.09, 1.39)**	1.26(1.12, 1.42)**	1.29(1.13, 1.46)**	1.28(1.13, 1.46)**
	Stunting-Underweight	1.17(1.08, 1.27)**	1.24(1.14, 1.34)**	1.25(1.16, 1.34)**	1.25(1.16, 1.34)**
	Wasting-Underweight	1.42(1.25, 1.61)**	1.37(1.23, 1.53)**	1.36(1.22, 1.51)**	1.36(1.22, 1.51)**
	Stunting-Wasting-Underweight	1.66(1.45, 1.89)**	1.51(1.35, 1.68)**	1.51(1.36, 1.68)**	1.51(1.36, 1.68)**
**Sex of the child**	Male	Ref	Ref	Ref	Ref
	Female	0.95(0.93, 0.99)**	0.96(0.94, 1.00)	0.97(0.94, 1.01)	0.97(0.94, 1.01)
**Child's age in months**	0–11	Ref	Ref	Ref	Ref
	12–23	1.40( 1.31, 1.49)**	1.30(1.23, 1.38)**	1.29( 1.22, 1.38)**	1.30(1.22, 1.38)**
	24–35	0.94(0.88, 1.00)	0.81(0.75, 0.87)**	0.81(0.75, 0.87)**	0.81(0.75, 0.87)**
	36–47	0.67(0.62, 0.74)**	0.59(0.55, 0.65)**	0.59(0.54, 0.64)**	0.59(0.54, 0.64)**
	48–59	0.52(0.47, 0.59)**	0.47(0.42, 0.51)**	0.45(0.41, 0.49)**	0.45(0.41, 0.50)**
**Birth order**	First	Ref	Ref	Ref	Ref
	2nd−4th	0.90(0.85, 0.95)**	0.94(0.87, 1.01)	0.96(0.91, 1.02)	0.96(0.91, 1.02)
	5th and above	0.82(0.78, 0.90)**	0.93(0.83, 1.03)	0.95(0.87, 1.03)	0.94(0.87, 1.03)
**Maternal age**	15–24	Ref	Ref	Ref	Ref
	25–34	0.82(0.80, 0.85)**	0.92(0.88, 0.96)**	0.92(0.88, 0.96)**	0.92(0.88, 0.97)**
	35–49	0.71(0.68, 0.76)**	0.84(0.79, 0.88)**	0.85(0.80, 0.89)**	0.85(0.80, 0.89)**
**Maternal educational status**	No	0.84(0.72, 0.98)*	0.99(0.87, 1.12)	0.93(0.79, 1.09)	0.93(0.79, 1.09)
	Primary education	1.03(0.92, 1.15)	1.12(1.01, 1.23)*	1.05(0.90, 1.22)	1.05(0.90, 1.22)
	Secondary education	1.08(0.99. 1.17)	1.09(1.02, 1.17)*	1.05(0.92, 1.18)	1.05(0.93, 1.19)
	Higher	Ref	Ref	Ref	Ref
**Marital status**	Not married	Ref	Ref	Ref	Ref
	Married/living with partner	0.89(0.84,.95)**	0.96(0.90, 1.02)	0.95(0.89, 1.01)	0.95(0.89, 1.01)
**Maternal working status**	Not working	Ref	Ref	Ref	Ref
	Working	1.14( 1.08, 1.20)**	1.21(1.14, 1.27)**	1.22(1.15, 1.29)**	1.22(1.15, 1.29)**
**Current breastfeeding status**	No	Ref	Ref		Ref
	Yes	1.02(0.96, 1.09)	0.79(0.76, 0.84)**	0.81(0.78, 0.84)**	0.81(0.78, 0.84)**
**Media exposure**	No	Ref	Ref	Ref	Ref
	Yes	1.23(1.16, 1.29)**	1.21(1.15, 1.27)**	1.23(1.17, 1.29)**	1.23(1.17, 1.29)**
**Water source**	Not improved	Ref	Ref	Ref	Ref
	Improved	1.04(0.98, 1.12)		1.03(0.98, 1.08)	1.03(0.99, 1.08)
**Toilet facility**	Not improved	Ref	Ref	Ref	Ref
	Improved	1.01(0.95, 1.07)		0.99(0.93, 1.06)	0.99(0.93, 1.06)
**Distance to a health facility**	Not a big problem	0.96(0.91, 1.01)		0.95(0.89, 1.00)	0.94(0.89, 1.00)
	Big problem	Ref		Ref	Ref
**Distance to water sources**	On-premises	0.99(0.90, 1.09)		0.97(0.89, 1.05)	0.96(0.88, 1.05)
	30 min and less	0.96(0.91, 1.02)		0.95(0.89, 1.01)	0.95(0.89, 1.01)
	Greater than 30 min	Ref		Ref	Ref
**Type of cooking fuel**	Unclean fuel	Ref		Ref	Ref
	Clean fuel	1.05(0.92, 1.19)		1.01(0.89, 1.13)	1.00(0.89, 1.12)
**Sex of household head**	Male	Ref		Ref	Ref
	Female	0.99(0.94, 1.06)		0.98(0.93, 1.03)	0.98(0.93, 1.03)
**Flooring materials**	Not improved	Ref		Ref	Ref
	Improved	1.01(0.93, 1.10)		0.98(0.93, 1.04)	0.98(0.93, 1.04)
**Roofing materials**	Not improved	Ref		Ref	Ref
	Improved	1.01(0.92, 1.11)		0.98(0.92, 1.04)	0.98(0.92, 1.04)
**Household wealth status**	Poorest	Ref		Ref	Ref
	Poorer	1.01(0.96, 1.07)		0.98(0.92, 1.05)	0.98(0.92, 1.04)
	Middle	1.05(0.97, 1.14)		0.98(0.89, 1.08)	0.98(0.89, 1.07)
	Richer	1.07(0.95, 1.20)		0.98(0.86, 1.12)	0.98(0.86, 1.10)
	Richest	1.02(0.88, 1.18)		0.91(0.77, 1.07)	0.90(0.77, 1.05)
**Handwashing station**	Not observed	Ref		Ref	Ref
	Observed	1.01(0.98, 1.05)		1.01(0.96, 1.07)	1.02(0.96, 1.07)
**Place of residence**	Rural	0.95(0.85, 1.07)			0.97(0.89, 1.07)
	Urban	Ref			Ref
**Community illiteracy level**	Low	Ref			Ref
	High	0.95(0.83, 1.07)			0.96(0.87, 1.06)
**Community poverty level**	Low	Ref			Ref
	High	0.97(0.86, 1.09)			1.04(0.96, 1.13)
**Year of survey**	Before covid	Ref			Ref
	During covid	0.78(0.55, 1.10)			0.78(0.56, 1.08)

## Discussion

Our study applied the CIAF approach and estimated variations in the effects of different standalone and coexisting patterns of undernutrition on common childhood infectious morbidity. The observed high prevalence of common infectious morbidity in SSA (30.02%) reflects its seriousness and ongoing public health concerns with far-reaching consequences among children under the age of five. This underscores the urgent need to uncover the root causes and implement effective, targeted interventions. The persistently high rates of infectious morbidity in SSA could attributed to factors such as widespread civil conflict, currently affecting about 21 countries in the region, resulting in displacement and healthcare system disruption; overcrowded living conditions and food insecurity.^38^, ^39^, ^40^

Our study found a statistically significant association between various forms of child undernutrition and common infectious morbidity supporting previous studies that have also documented such significant association.^10^, ^41^, ^42^ The increased risk of infectious morbidity among undernourished children is predictable, as undernutrition generally weakens the body's immune defenses and loosens the epithelial barrier, making children more susceptible to various infectious conditions.^11^ Most importantly, this study shed light on a significant variation in the link between standalone, double, and triple forms of undernutrition and common infectious morbidity in children under five. Specifically, children with coexisting undernutrition were most affected by common infectious morbidity, with the odds peaking among children facing the coexistence of stunting-wasting-underweight. Our findings are supported by a few studies that demonstrated a higher odd of common infectious morbidity among children with coexisting undernutrition.^23^, ^24^, ^29^ On the other hand, standalone forms of undernutrition showed no or minimal association with common infectious morbidity. This might be because children with standalone undernutrition still preserve some immune capacity or metabolic reserves, enabling better resistance to and recovery from infections. However, coexistence of different forms of undernutrition on the same child might severely compromise immune function and deplete essential nutrients, placing these children at an extra high risk of infectious morbidity.^43^

Based on the findings of this study we could suggest that children who are highly susceptible to infectious morbidity should be prioritised for urgent care and immediate interventions such as prompt diagnosis and treatment of the underlying causes and nutritional supports. Focusing on these most vulnerable groups of children would ensure that resources and efforts are mobilized toward where they are most needed in the resource-limited setting and aid in effectively mitigating the burden of infectious morbidity. On the other hand, children with a relatively low odds of infectious conditions, such as stunting only and wasting only, can be deprioritized from nutrition interventions, supplementation, and treatment priorities. This targeted action might help accelerate the sustainable aim of ending preventable deaths from diarrhea and ARI.^3^ Moreover, the nutrient and energy requirements for children with standalone and coexisting undernutrition might be different. It is crucial to update the nutrient contents, amounts and duration of therapeutic feeding formula, to reach children with the complex double and triple undernutrition. In general, there is a need for distinct nutritional approaches for children of high versus low infectious disease vulnerability, unlike current approaches that do not account for these differences in infection risk.^44^

The age of the child was another essential determinant of common infectious morbidity, where children aged 12–23 months were associated with a higher odd of infectious morbidity. This is likely because children in this age group start crawling, walking, and exploring their immediate environment, interact with others, and are more prone to picking up infectious agents.^45^ Children in this age group are also on complementary feeding, which increases their risk of exposure to contaminated agents.^46^ However, as children grow older, their immunity strengthens, and they learn how to interact more effectively with their environment by avoiding contacts with unclean things.^45^ This could explain the lower odds of experiencing common infectious morbidity among older children aged 24–59 months.

Furthermore, our study found that children born to mothers aged 25 years and above had a reduced odds of experiencing common infectious morbidity compared to children born to young mothers (15–24 years). Our finding is consistent with previous reports.^47^, ^48^ However, other studies reported no association between maternal age and common childhood infectious morbidity.^34^, ^49^ The protective effect of increased maternal age on common infectious morbidity can be justified because older mothers are more likely to be educated, better informed about proper child-feeding, and are more experienced in childcare practice compared to young mothers.^48^

Children of working mothers were more likely to experience common infectious morbidity compared to children of non-working mothers. This can be explained by the fact that working mothers often have less time to devote to caregiving and providing proper feeding to their children, including breastfeeding, due to work-related commitments.^50^ Previous studies also reported that children of working mothers were at an increased risk of common infectious morbidity.^37^, ^50^

In this study, the likelihood of common childhood infectious morbidity was found to be higher among mothers having media exposure. In contrast, previous studies reported maternal media exposure as a protective factor against common childhood illnesses among under-five children.^48^, ^51^ The DHS method of assessing media exposure lacks specificity, as it only captures the frequency of media engagement without detailing the content encountered.^31^ As the media exposure variable in the DHS data is collected implicitly, mothers might have equal chance of being exposed to harmful contents of the media such as the frequent advertisements and promoting of junk food, processed snacks, and sugary beverages which are considered poor in dietary diversity and nutritional value for the growing children.^52^ Based on our findings, we can conclude that media exposure alone does not always lead to positive outcomes for child health. Instead, the content and the specific message conveyed and how it is received also matter.

This study has several strengths that enhance the validity of the findings. The large sample size from nationally representative DHS of SSA countries increases the study's statistical power in identifying determinants of common infectious morbidity. Additionally, this study used hierarchical modeling to account for clustering at community, and country levels, enhancing the reliability of the estimates obtained. Moreover, using the disaggregated patterns of CIAF, this study provided a more comprehensive understanding of the association of child undernutrition with common infectious morbidity and identified the most vulnerable groups.

However, this study has some limitations inherent to the data used, that should be considered when using the findings.^53^ Firstly, data on common infectious morbidity relied on maternal self-reports rather than clinical assessments. As a result, recall bias might significantly affect our results. Secondly, the causal relationship between common infectious morbidity, child undernutrition, and other covariates cannot be established as we used cross-sectional survey data. Although the two-week recall period for infectious morbidity and the chronic nature of undernutrition suggest that undernutrition likely precedes morbidity, the possibility of reverse association cannot be entirely excluded. This is particularly important given the well-established bidirectional relationship between undernutrition and infectious diseases. Therefore, longitudinal studies are needed to more accurately establish temporal associations between undernutrition and common infectious morbidity, particularly the varying impacts of standalone, double and triple undernutrition on childhood infectious morbidity.

## Conclusions

Our study highlighted the high prevalence of common infectious morbidity among children under five in SSA. Children with any of the coexisting forms of undernutrition were at an increased odd of infectious morbidity, with those experiencing triple undernutrition carrying the most significant odds of infectious morbidity. However, standalone forms of undernutrition showed no or minimal association with infectious morbidity. This indicates that children with coexisting undernutrition are the most likely to benefit from nutrition and other child survival interventions. Therefore, to accelerate the reduction of high burdens of common infectious morbidity, decision-makers and policymakers should introduce tailored nutrition interventions targeting children with coexisting undernutrition. Furthermore, adopting age-specific infectious protection, prevention, and treatment strategies may be crucial in effectively reducing common childhood infectious morbidity in SSA.

## Abbreviations

AOR: Adjusted odds ratio, ARI: Acute respiratory tract infection, AIC: Akaike’s Information Criterion, ACT: Australian capital territory, BIC: Bayesian Information Criterion, CIAF: Composite index of anthropometric failure, CI: Confidence interval, CSB + + : Corn-Soya Blends Plus, DHS: Demographic health survey, GAPPD: Global Action Plan for the Prevention and Control of Pneumonia and Diarrhea, ICC: Intraclass Correlation Coefficient, LNS: Lipid-based nutrient supplements, LMICs: Low- and middle-income countries, MOR: Median Odds Ratio, PCV: Proportional Change in Variance, RUTF: Ready-to-use therapeutic food, SSA: Sub-Sahara Africa, SDGs: Sustainable development goals, UN: United nation, UNICEF: United Nations International Children's Emergency Fund, VIF: Variance inflation factors, WHO: World health organization.

## CRediT authorship contribution statement

**Itismita Mohanty:** Writing – review & editing, Supervision, Software, Methodology, Formal analysis, Data curation, Conceptualization. **Zelalem Mengesha:** Writing – review & editing, Supervision, Software, Methodology, Formal analysis, Data curation, Conceptualization. **Theo Niyonsenga:** Writing – review & editing, Supervision, Software, Methodology, Formal analysis, Data curation, Conceptualization. **Misganaw Gebrie Worku:** Writing – review & editing, Writing – original draft, Software, Methodology, Formal analysis, Data curation, Conceptualization.

## Consent for publication

Not applicable

## Ethical approval and consent to participant

This study used publicly available, de-identified DHS data. The DHS team strictly considered informed consent and ethical issues during the data collection process. Ethical approval was granted by the University of Canberra Human Research Ethics Committee (approval no. 13640).

## Funding

No funding was obtained for this study.

## Declaration of Competing Interest

All authors declare that they have no competing interests.

## Data Availability

The authors do not have permission to share data.
